# An Anoikis‐Related Gene Signature Associated With Stromal Remodeling and Clinical Outcome in Gastric Cancer

**DOI:** 10.1111/cbdd.70411

**Published:** 2026-09-30

**Authors:** Zhijun Fu, Lin Sun, Xing Wang, Biao Cheng, Ying Huang, Yuzhu Tang, Zhihua Jin

**Affiliations:** ^1^ Department of General Surgery Wusheng County People's Hospital (Wusheng Hospital Affiliated Hospital of North Sichuan Medical College) Guang'an Sichuan China; ^2^ Department of Gastroenterology Chongqing Southeast Hospital Chongqing China; ^3^ Department of Oncology People's Hospital of Yilong County Nanchong Sichuan China; ^4^ Department of Oncology, Xinqiao Hospital Army Medical University Chongqing China; ^5^ Department of Gastrointestinal Surgery People's Hospital of Fengjie County Chongqing China

**Keywords:** anoikis‐related genes, endothelial‐to‐mesenchymal transition, gastric cancer, single‐cell RNA sequencing, tumor microenvironment

## Abstract

Gastric cancer (GC) remains a highly aggressive malignancy with poor prognosis. Anoikis resistance is closely associated with tumor progression and metastasis, but the prognostic and functional relevance of anoikis‐related genes (ARGs) in GC remains incompletely understood. Public transcriptomic, clinical, somatic mutation, and single‐cell RNA sequencing (scRNA‐seq) datasets were integrated to identify differentially expressed ARGs, develop an ARG‐based risk model, and investigate its associations with tumor microenvironment (TME) features. In vitro experiments were performed to assess the effects of selected candidate genes on GC cell migration and invasion. Forty‐five differentially expressed ARGs were identified in The Cancer Genome Atlas Stomach Adenocarcinoma (TCGA‐STAD) cohort. An ARG‐based risk model was developed using the TCGA‐STAD cohort and evaluated in the GSE84437 cohort. The model was associated with overall survival and provided potential value for risk stratification, although its discriminative performance was modest. High‐risk patients showed distinct immune, stromal, and somatic mutational features. Single‐cell analysis suggested that ARG‐related transcriptional programs were associated with stromal remodeling and endothelial‐to‐mesenchymal transition (EndMT)‐associated features in the GC microenvironment. Among the core ARGs, EEF1A2 and TAGLN showed prognostic relevance and higher protein expression in GC tissues. Small interfering RNA (siRNA)‐mediated knockdown of either gene reduced the migration and invasion of HGC‐27 cells. These findings indicate that ARG‐related molecular features are associated with GC prognosis and immune‐stromal characteristics, while EEF1A2 and TAGLN may represent candidate genes related to GC cell migration and invasion.

## Graphical abstract

An externally validated anoikis‐related gene signature stratifies gastric cancer prognosis and is associated with immune‐stromal remodeling. EEF1A2 and TAGLN were further validated as candidate genes involved in gastric cancer cell migration and invasion.

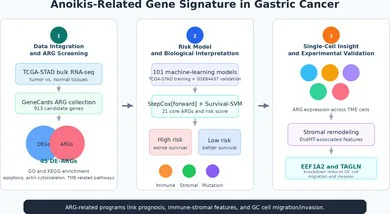

## 1 Introduction

Gastric cancer (GC) remains a major global health burden, with approximately 969,000 new cases and 660,000 deaths worldwide in 2022 (Patel et al. 2026; Bray et al. 2024). Despite advances in surgery, chemotherapy, radiotherapy, immunotherapy, and targeted therapy (Joshi and Badgwell 2021), recurrence and metastasis remain major challenges in advanced GC. Identifying molecular features associated with GC progression and prognosis is therefore important for patient stratification and therapeutic management. Recent transcriptomic studies have highlighted that GC progression is shaped by both tumor‐cell‐intrinsic alterations and interactions with the tumor microenvironment (TME), including immune, stromal, and endothelial components.

Anoikis is a specialized form of programmed cell death triggered by loss of extracellular matrix attachment or disruption of cell–cell adhesion. Anoikis resistance allows detached tumor cells to survive after loss of adhesion and may facilitate migration, invasion, and metastatic colonization, thereby contributing to tumor progression and poor prognosis in multiple malignancies, including GC (Taddei et al. 2012; Anderson and Simon 2020; Wang et al. 2025). These progression‐related phenotypes are closely linked to TME remodeling, particularly alterations in stromal and endothelial compartments. Endothelial‐to‐mesenchymal transition (EndMT), through which endothelial cells acquire mesenchymal‐like features, has been implicated in stromal remodeling and fibroblast‐like cellular states within tumors (Lebas et al. 2024; Ciszewski et al. 2021; Potenta et al. 2008). Given the close links among anoikis resistance, loss of adhesion, migration and invasion, and stromal remodeling, ARG‐related transcriptional programs may be associated with EndMT‐associated cellular states in GC; however, this relationship remains incompletely understood (Chhabra and Weeraratna 2023; Wu et al. 2021).

In this study, we integrated bulk transcriptomic, whole‐exome sequencing (WES)‐derived somatic mutation, single‐cell RNA sequencing (scRNA‐seq), and in vitro experimental data to characterize ARG‐related molecular features in GC. Differentially expressed ARGs were identified from the Cancer Genome Atlas Stomach Adenocarcinoma (TCGA‐STAD) cohort, and multiple machine‐learning algorithm combinations were evaluated to construct an externally validated ARG‐based risk model. We then examined the associations of this model with patient survival, immune infiltration, stromal features, and somatic mutational landscapes. Given the utility of scRNA‐seq for resolving TME heterogeneity and cell type‐specific transcriptional programs (Aran 2023), single‐cell analysis was performed to characterize the cellular distribution of ARG‐related transcriptional programs and their potential association with stromal remodeling and EndMT‐associated cellular states. Finally, EEF1A2 and TAGLN were selected for in vitro validation to assess their effects on GC cell migration and invasion.

## 2 Materials and Methods

### 2.1 Data Acquisition

Transcriptomic and clinical data for GC were obtained from public databases. RNA sequencing data and clinical information for the TCGA‐STAD cohort, including 412 tumor samples and 36 normal gastric tissue samples, were downloaded from the UCSC Xena platform (https://xena.ucsc.edu/). Clinical variables included survival status, age, T stage, N stage, M stage, and tumor grade. The GSE84437 dataset, comprising 433 GC samples, was obtained from the Gene Expression Omnibus (GEO) database and used for external validation. scRNA‐seq data from GSE162115, including intratumoral and adjacent tissue samples from patients with GC, were used to characterize ARG distribution, stromal cell subpopulations, and EndMT‐associated molecular features. ARGs were retrieved from GeneCards using the keyword “anoikis‐related,” yielding 913 candidate genes. No stringent relevance score threshold was applied during initial retrieval to maximize the inclusion of potentially relevant ARGs; downstream differential expression, survival, and machine‐learning‐based feature selection analyses were then used to improve biological specificity. Immunohistochemical images were obtained from the Human Protein Atlas (HPA; https://www.proteinatlas.org/). WES‐derived somatic mutation data for TCGA‐STAD were obtained from cBioPortal (https://www.cbioportal.org/) (de Bruijn et al. 2023) and used to compare mutational landscapes between the high‐ and low‐risk groups.

### 2.2 Construction and Independent Evaluation of the ARG‐Based Risk Model

Differentially expressed genes (DEGs) between GC and normal gastric tissues in the TCGA‐STAD cohort were identified using thresholds of |log_2_ fold change| ≥ 2 and adjusted *p* value < 0.05. Differentially expressed ARGs were obtained by intersecting these DEGs with ARGs retrieved from GeneCards. The TCGA‐STAD expression matrix and survival data were used for model construction, and GSE84437 was used for independent evaluation. Ten machine‐learning algorithms, including Elastic Net (Enet), Ridge regression, partial least squares regression for Cox models (plsRcox), Stepwise Cox regression, least absolute shrinkage and selection operator (Lasso), survival support vector machine (Survival‐SVM), random survival forest (RSF), supervised principal components (SuperPC), CoxBoost, and generalized boosted regression modeling (GBM), were integrated to generate 101 algorithm combinations. A 10‐fold cross‐validation strategy was applied in TCGA‐STAD for feature selection and model training. Model performance was assessed using the concordance index (C‐index), and the top five models ranked by mean C‐index across the training and validation cohorts were compared. StepCox [forward] + Survival‐SVM was selected for subsequent analyses because it showed relatively consistent C‐index values across the two cohorts and allowed calculation of individual risk scores. Patients were stratified into high‐ and low‐risk groups according to the median risk score within each cohort. Kaplan–Meier survival analysis, univariate and multivariate Cox regression analyses, and comparisons with published prognostic signatures were performed to assess the prognostic association and potential value of the model for risk stratification. Gene Ontology (GO) and Kyoto Encyclopedia of Genes and Genomes (KEGG) enrichment analyses of differentially expressed ARGs were performed using clusterProfiler and visualized with enrichplot. Terms with an adjusted *p* value < 0.05 were considered significant.

### 2.3 Immune Infiltration Analysis

To investigate the relationship between the ARG‐based risk model and the TME in GC, stromal, immune, and ESTIMATE scores were calculated for each TCGA‐STAD sample using the ESTIMATE algorithm (https://bioinformatics.mdanderson.org/estimate/). WES‐derived somatic mutation data were analyzed and visualized using the maftools R package to compare mutational landscapes between the high‐ and low‐risk groups. The CIBERSORT algorithm was used to estimate the relative proportions of 22 immune cell populations in the TCGA‐STAD cohort. Representative immune‐related genes and cytokine‐related genes were compared between the two risk groups, and correlation analysis was performed to evaluate the association between the ARG‐based risk score and CD86 expression.

### 2.4 Single‐Cell RNA‐Seq Analysis

scRNA‐seq data from GSE162115 were analyzed using the Seurat package (v4.3.0) in R. Quality control was performed by examining detected genes (nFeature_RNA), total RNA counts (nCount_RNA), and other basic sequencing metrics across samples, and potential doublets were identified using DoubletFinder and removed. After normalization and identification of highly variable genes, principal component analysis (PCA) was performed for dimensionality reduction. Cell clustering was conducted using the FindNeighbors and FindClusters functions in Seurat, and cell distributions were visualized using t‐distributed stochastic neighbor embedding (t‐SNE). Cell types were annotated based on canonical marker genes from CellMarker 2.0 and published literature. To evaluate transcriptional heterogeneity, inferCNV analysis was performed using T cells, B cells, and dendritic cells (DCs) as reference populations. inferCNV was used to assess chromosome‐scale coordinated gene‐expression shift patterns rather than true DNA copy number variations (CNVs); therefore, the results were interpreted as indicators of transcriptional heterogeneity. Based on inferCNV results and inferCNV score distributions, fibroblasts were subdivided into distinct subpopulations. Pseudotime trajectory analysis was performed using Monocle 3 to investigate potential transcriptional‐state relationships between endothelial cells and fibroblast subpopulations. ARG scores and EndMT scores were calculated using single‐sample gene set enrichment analysis (ssGSEA), and their correlations were assessed using Pearson or Spearman correlation analysis according to data distribution. The EndMT score was generated based on previously reported endothelial and mesenchymal marker genes related to EndMT, including CDH5, PECAM1, CLDN5, VWF, TEK, ACTA2, VIM, and FN1 (Xu and Kovacic 2023).

### 2.5 Cell Culture, Transfection, and Migration/Invasion Assays

The human GC cell line HGC‐27 was obtained from Wuchuan Biotechnology (China) in 2025, with authentication information provided by the supplier. Independent short tandem repeat (STR) profiling was not performed; however, no misidentification record for HGC‐27 was found in the International Cell Line Authentication Committee (ICLAC) Register of Misidentified Cell Lines or the Cellosaurus database at the time of analysis (Bairoch 2018). Routine mycoplasma testing was not performed, but cells were maintained under sterile conditions and used within a limited number of passages. Cells were cultured in RPMI‐1640 medium containing 10% fetal bovine serum and 1% penicillin–streptomycin at 37°C with 5% CO_2_. At approximately 70% confluence, cells were transfected with small interfering RNAs (siRNAs) targeting EEF1A2 or TAGLN (Beijing Aojing Biotechnology Co. Ltd.), and knockdown efficiency was assessed 72 h after transfection. For quantitative real‐time PCR (qRT‐PCR), total RNA was extracted using Total RNA Extraction Reagent (ABclonal, USA, RK30129), and complementary DNA (cDNA) was synthesized using the PrimeScript RT Reagent Kit (TaKaRa, Japan, RR037A). qRT‐PCR was performed on a Bio‐Rad CFX96 system (Bio‐Rad, USA) using SYBR Premix Ex Taq II (TaKaRa, Japan, RR420A). β‐actin was used as the internal control, and relative gene expression was calculated using the $2^{-\Delta \Delta C_{t}}$ method. All experiments were performed in triplicate. The primer sequences were as follows: β‐actin forward, CATGTACGTTGCTATCCAGGC and reverse, CTCCTTAATGTCACGCACGAT; TAGLN forward, AGTGCAGTCCAAAATCGAGAAG and reverse, CTTGCTCAGAATCACGCCAT; EEF1A2 forward, GAAGACCCACATCAACATCGT and reverse, CTCCGCATTTGTAGATGAGGTG. For Western blotting, proteins were extracted using a total protein extraction kit, quantified by the bicinchoninic acid (BCA) method, separated by sodium dodecyl sulfate‐polyacrylamide gel electrophoresis (SDS‐PAGE), and transferred onto polyvinylidene fluoride (PVDF) membranes. After blocking with 5% nonfat milk, membranes were incubated with primary antibodies against EEF1A2 (ab227275, Abcam), TAGLN (ab14106, Abcam), and β‐actin (BM0627, BOSTER) overnight at 4°C, followed by horseradish peroxidase (HRP)‐conjugated secondary antibodies at 37°C for 2 h. Signals were visualized using enhanced chemiluminescence (ECL) and detected with a chemiluminescence imaging system. For Transwell migration assays, approximately 5 × 10^4^ HGC‐27 cells in serum‐free medium were seeded into the upper chambers of 8‐μm Transwell inserts, with RPMI‐1640 medium containing 10% fetal bovine serum in the lower chamber. After incubation at 37°C for 24 h, migrated cells were fixed with 4% paraformaldehyde, stained with hematoxylin and eosin, photographed, and counted in randomly selected fields. For invasion assays, the upper chambers were pre‐coated with Matrigel (Corning, USA), and invading cells were quantified using the same procedure.

### 2.6 Statistical Analysis

Statistical analyses were performed using R software (v4.2.1) and GraphPad Prism 8. Continuous variables were summarized as mean ± standard deviation or median with interquartile range, as appropriate. Kaplan–Meier curves were compared using the log‐rank test, and Cox regression models were used to evaluate prognostic factors. Between‐group comparisons were performed using Student's *t*‐test or the Wilcoxon rank‐sum test, as appropriate. Correlations were assessed using Pearson or Spearman correlation analysis according to data distribution. Adjusted *p* values were used for enrichment analyses, and a two‐sided *p* value < 0.05 was considered statistically significant unless otherwise indicated.

## 3 Results

### 3.1 Screening and Functional Characterization of Differentially Expressed ARGs

A total of 913 ARGs were retrieved from GeneCards (Table S1). Differential expression analysis identified DEGs between GC and normal gastric tissues in the TCGA‐STAD cohort (Figure 1A), and intersection with the ARG set yielded 45 differentially expressed ARGs (Figure 1B). KEGG and GO enrichment analyses were performed to explore their biological functions (Figure 1C,D; Tables S2 and S3). KEGG analysis showed enrichment in apoptosis, regulation of actin cytoskeleton, insulin resistance, type II diabetes mellitus, adipocytokine signaling, and IL‐17 signaling pathways, whereas GO analysis indicated enrichment in apoptosis‐related processes, vesicle‐ and extracellular matrix‐related components, endopeptidase activity, and Toll‐like receptor binding. These findings suggest that differentially expressed ARGs are involved in GC progression‐related biological processes and support their use for subsequent risk model construction.

**FIGURE 1:**
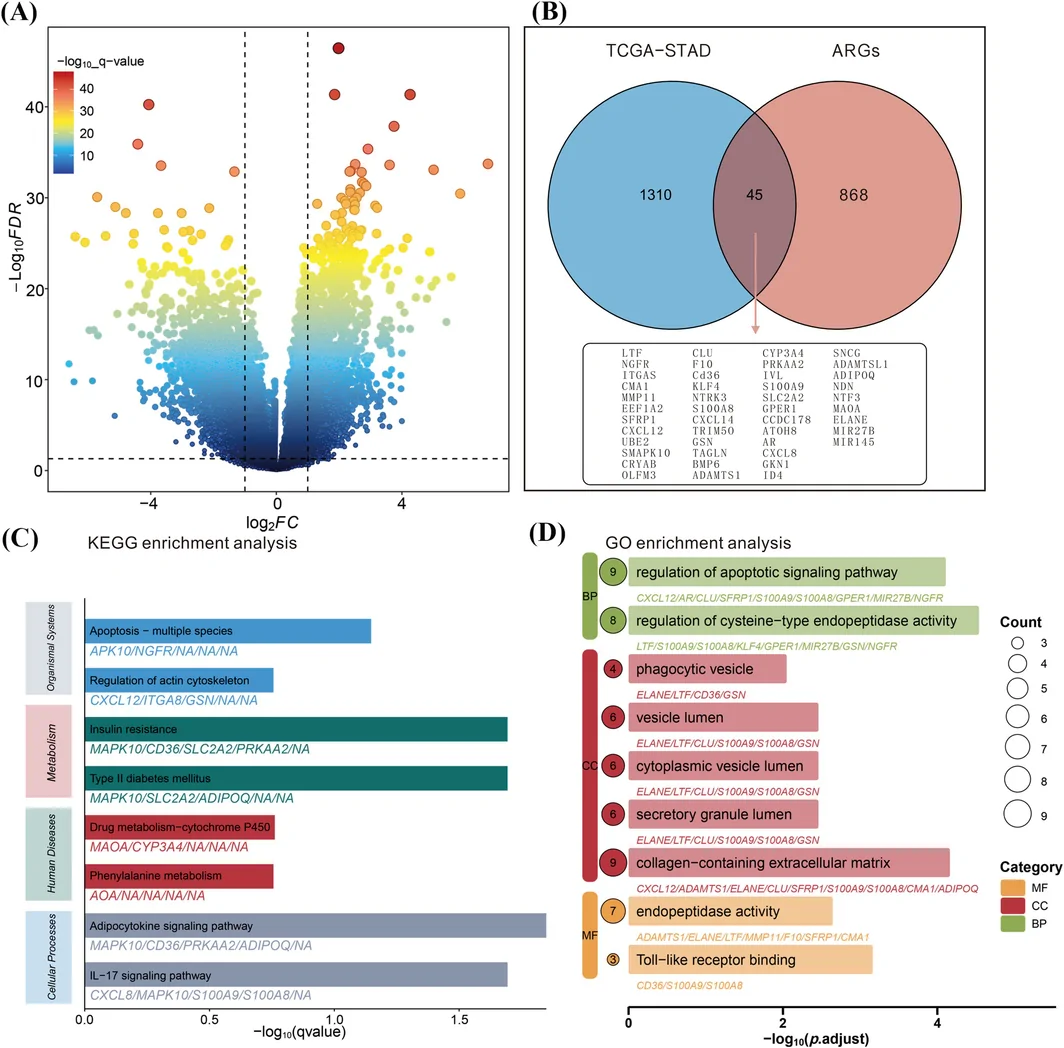
Identification and enrichment analysis of differentially expressed ARGs in GC. (A) Volcano plot of DEGs in the TCGA‐STAD cohort. (B) Overlap between TCGA‐STAD DEGs and ARGs. (C) KEGG enrichment analysis of differentially expressed ARGs. (D) GO enrichment analysis of differentially expressed ARGs.

### 3.2 Construction and External Validation of the ARG‐Based Risk Model

Using the 45 differentially expressed ARGs, we constructed and externally validated an ARG‐based risk model. Model training was performed in the TCGA‐STAD cohort, with external validation in the GSE84437 cohort (Figure 2A). Based on the average C‐index across both cohorts, the five best‐performing models were SuperPC, StepCox[forward] + SuperPC, RSF + SuperPC, Survival‐SVM, and StepCox[forward] + Survival‐SVM (Figure 2B; Table S4). Although the overall C‐index values were moderate, StepCox[forward] + Survival‐SVM showed highly consistent performance in TCGA‐STAD and GSE84437, with C‐index values of 0.580 and 0.578, respectively. Because this model also enabled gene‐level feature selection and individual risk‐score calculation, it was selected for subsequent risk stratification and biological interpretation. The final model identified 21 core ARGs, including PDK4, NGFR, CMA1, EEF1A2, SFRP1, MAPK10, CRYAB, OLFM3, CD36, NTRK3, TRIM50, TAGLN, BMP6, ADAMTS1, PRKAA2, AR, ID4, SNCG, ADAMTSL1, ADIPOQ, and NDN. Correlation analysis revealed distinct co‐expression patterns among these genes (Figure 2C), and univariate Cox regression analysis showed that several signature genes were associated with overall survival (Figure 2D). Patients were stratified into high‐ and low‐risk groups according to the median risk score within each cohort. Kaplan–Meier analysis showed significantly worse overall survival in the high‐risk group in both TCGA‐STAD (HR = 1.56, 95% CI = 1.15–2.13, *p* = 0.005) and GSE84437 (HR = 1.73, 95% CI = 1.32–2.28, *p* < 0.001) (Figure 2E,F). Compared with published prognostic signatures, the ARG‐based risk model showed comparable C‐index values in the evaluated cohorts (Figure 2G,H). Multivariate Cox regression further showed that the ARG‐based risk score remained significantly associated with overall survival after adjustment for available clinical factors (HR = 2.187, 95% CI = 1.234–3.874, *p* = 0.007; Table S5).

**FIGURE 2:**
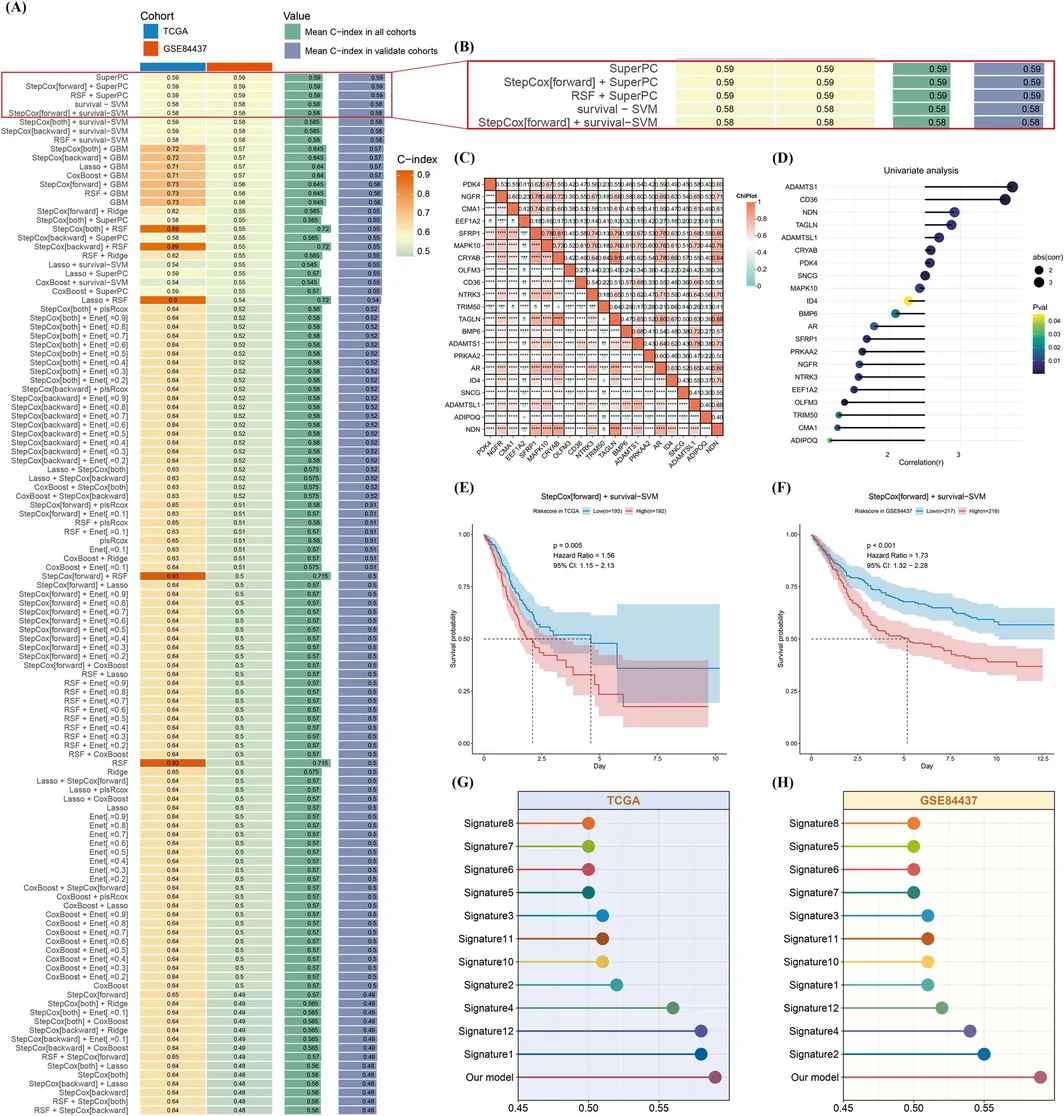
Construction and validation of the ARG‐based risk model. (A) C‐index heatmap of 101 machine‐learning algorithm combinations in the TCGA‐STAD and GSE84437 cohorts. (B) Top five algorithm combinations ranked by mean C‐index. (C) Pairwise correlation matrix of genes in the ARG‐based risk model. (D) Univariate Cox regression analysis of model genes. (E, F) Kaplan–Meier curves for high‐ and low‐risk groups in the TCGA‐STAD and GSE84437 cohorts. (G, H) C‐index comparison between the ARG‐based risk model and published prognostic signatures in the TCGA‐STAD and GSE84437 cohorts.

### 3.3 Immune and Genomic Features Associated With the ARG‐Based Risk Model

To interpret the biological features associated with the ARG‐based risk model, we compared immune, stromal, and somatic mutational characteristics between the high‐ and low‐risk groups. ESTIMATE analysis showed significant differences in ESTIMATE, immune, and stromal scores, suggesting distinct TME features across risk strata (Figure 3A–C). WES‐derived somatic mutation analysis revealed different mutational landscapes between the two groups, indicating potential genomic heterogeneity associated with the risk model (Figure 3D,E). Several immune‐related genes, including AHSA1, CD163, CD37, and IL2RB, and cytokine‐related genes, including IL10, IL13, IL15, and IL16, were differentially expressed between groups (Figure 3F,G). CIBERSORT analysis revealed differences in multiple immune cell subsets, including naïve B cells, activated CD4 memory T cells, follicular helper T cells, resting NK cells, monocytes, M2 macrophages, activated DCs, mast cells, and neutrophils (Figure 3H). In addition, the ARG‐based risk score was positively correlated with CD86 expression (*r* = 0.33, *p* < 0.001; Figure 3I). These findings reveal that the ARG‐based risk model is associated with distinct immune, stromal, and somatic mutational features in GC.

**FIGURE 3:**
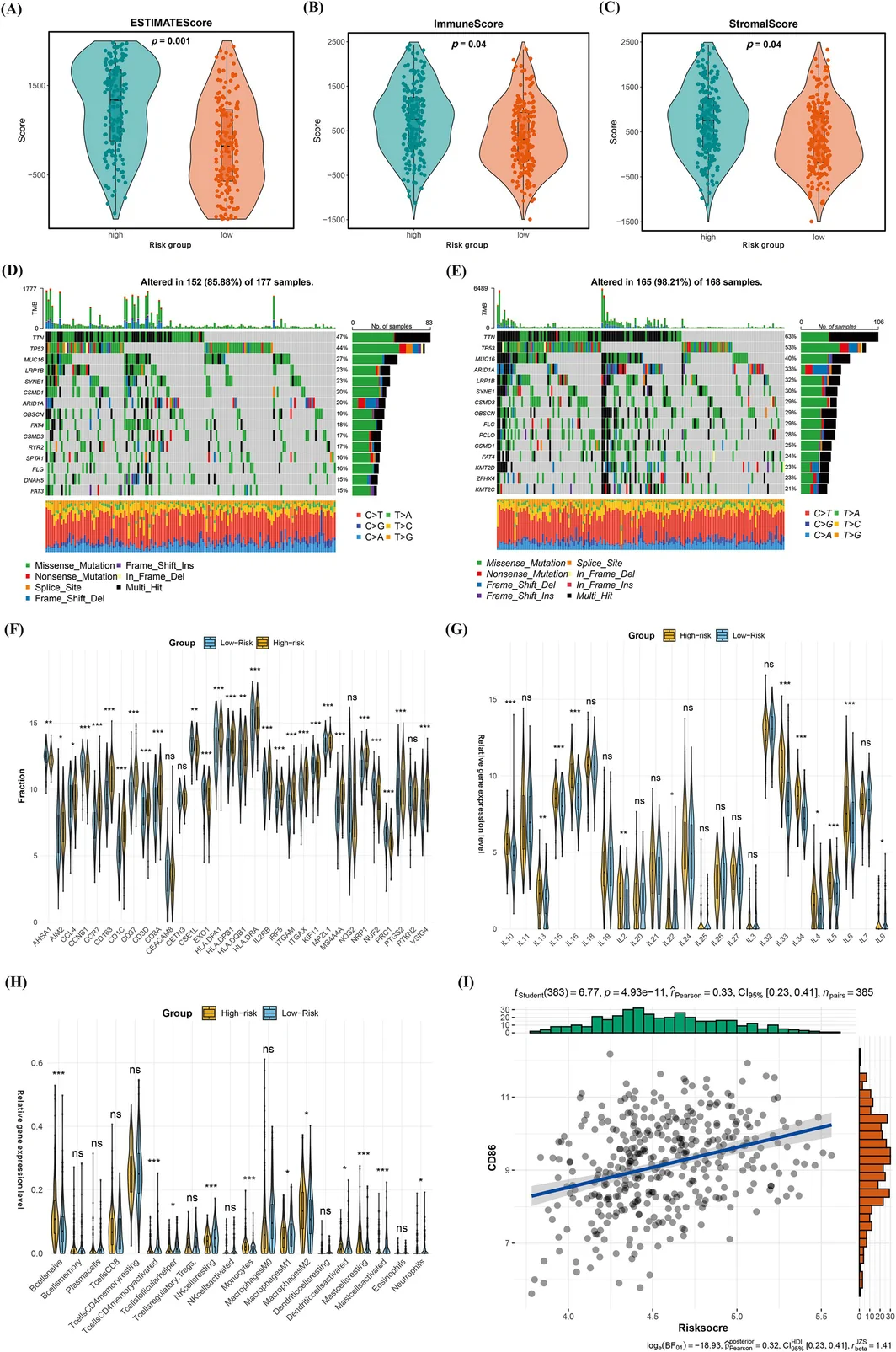
Immune and genomic features associated with the ARG‐based risk model. (A–C) Violin plots showing ESTIMATE, immune, and stromal scores in the high‐ and low‐risk groups; individual observations are shown as dots. (D, E) Somatic mutation landscapes in the high‐risk group (*n* = 177) and low‐risk group (*n* = 168). (F–H) Violin plots showing immune‐related gene expression, cytokine‐related gene expression, and CIBERSORT‐estimated immune cell composition, respectively. (I) Correlation between the ARG‐based risk score and CD86 expression; Pearson correlation analysis was used (*n*_pairs = 385). The Wilcoxon rank‐sum test was used for panels A–C and F–H. No error bars are shown. *p < 0.05, **p < 0.01, and ***p < 0.001; ns, not significant.

### 3.4 Single‐Cell Analysis Reveals Associations Between ARG‐Related Transcriptional Programs, Stromal Remodeling, and EndMT‐Associated Features in GC

To investigate the cellular context of ARG‐related molecular features, we analyzed the GSE162115 scRNA‐seq dataset. Quality control showed comparable distributions of detected genes and transcript counts, a low predicted doublet fraction, and limited cell‐cycle effects, supporting downstream analyses (Figure S1). After dimensionality reduction and clustering, 35 cell clusters were annotated into seven major cell types, including T cells, B cells, NK cells, DCs, endothelial cells, fibroblasts, and monocytes/macrophages (Figure 4A–C). To evaluate transcriptional heterogeneity within the TME, inferCNV analysis was performed using immune‐cell populations as reference cells. Endothelial cells and fibroblasts showed more pronounced chromosome‐scale expression shift patterns and higher inferCNV scores than immune‐cell populations, suggesting increased transcriptional heterogeneity within these stromal compartments (Figure 4D,E). Fibroblasts were further subdivided into 10 subclusters (F0–F9), among which F2, F3, F4, F8, and F9 displayed relatively elevated inferCNV scores (Figure 4F,G). Pseudotime analysis showed a trajectory involving endothelial cells and fibroblast subpopulations, with endothelial cells mainly distributed at earlier trajectory states and several fibroblast subpopulations at later states (Figure 4H). Core ARGs showed heterogeneous expression patterns across cell populations, with representative genes including PDK4, NGFR, EEF1A2, SFRP1, MAPK10, CRYAB, CD36, and TAGLN displaying cell type‐specific distributions (Figure 4I). Correlation analysis showed a positive association between ARG score and EndMT score, suggesting that ARG‐related transcriptional programs may be linked to EndMT‐associated features (Figure 4J). Dynamic expression analysis further showed that CD36 gradually decreased, whereas CRYAB and SFRP1 increased along the inferred stromal‐state trajectory (Figure S2B). Together, these findings suggest that ARG‐related transcriptional programs may be associated with stromal remodeling and EndMT‐associated cellular states in GC.

**FIGURE 4:**
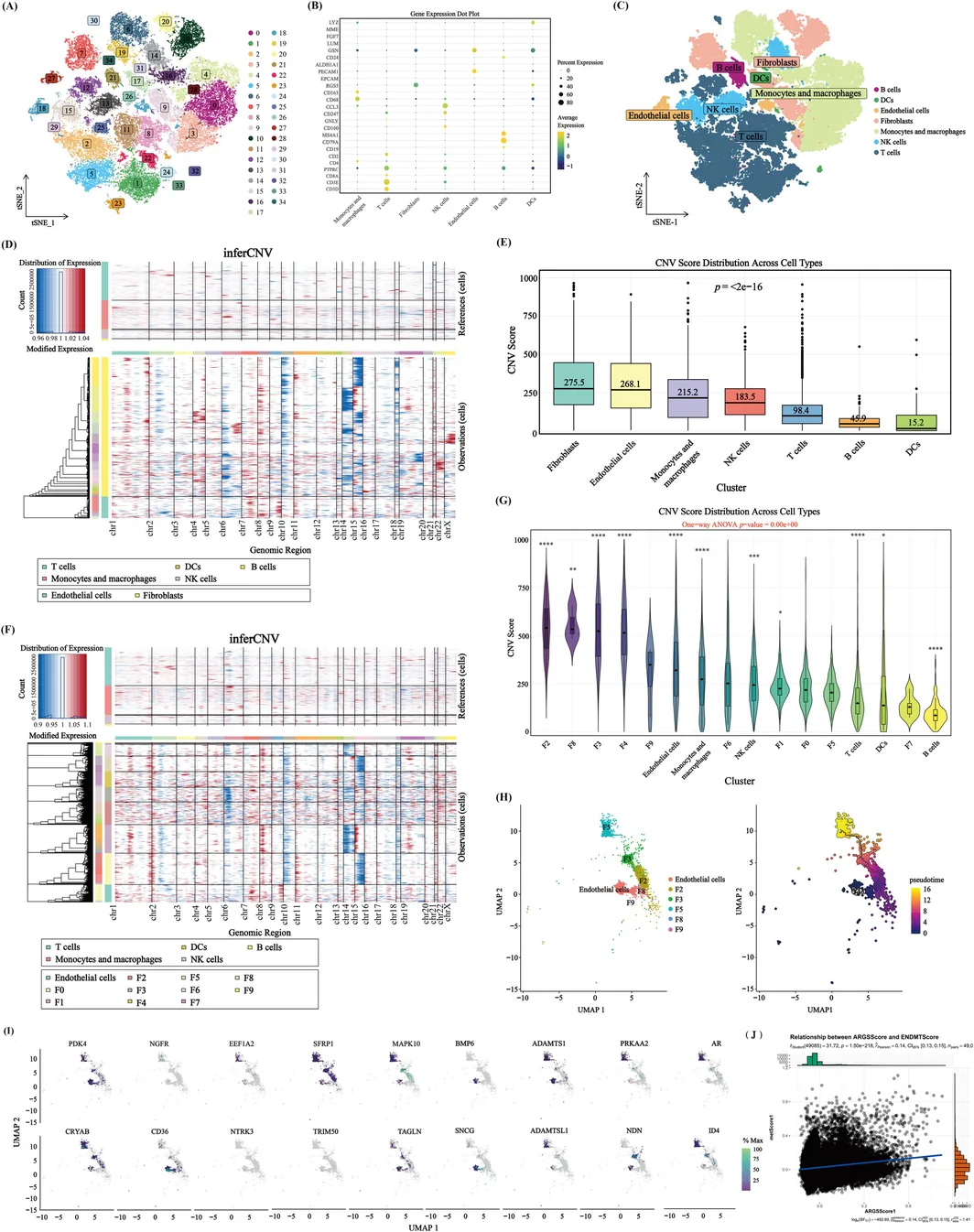
Single‐cell analysis of stromal remodeling and ARG‐related features in GC. (A) t‐SNE plot of 35 cell clusters. (B) Dot plot of canonical marker genes; dot size indicates the percentage of expressing cells and dot color indicates average expression. (C) t‐SNE plot of annotated cell populations. (D, F) inferCNV heatmaps of major cell populations and fibroblast subclusters, respectively. (E) Boxplots of inferCNV scores across major cell populations. (G) Violin plots of inferCNV scores across fibroblast subclusters; one‐way analysis of variance was used as indicated in the panel. (H) UMAP and pseudotime trajectory of endothelial cells and fibroblasts. (I) Feature plots of representative ARGs. (J) Correlation between ARG and EndMT scores; Spearman rank correlation analysis was used. Statistical significance was assessed using the statistical procedures applied in the original analyses, and *p* values are shown in the corresponding panels. No error bars are shown. ns, not significant; *p < 0.05, **p < 0.01, ***p < 0.001, and ****p < 0.0001.

### 3.5 Functional Validation of EEF1A2 and TAGLN in GC Cells

To evaluate the clinical and biological relevance of core ARGs, we examined their prognostic associations and protein expression patterns in GC. Kaplan–Meier analysis showed that ADAMTS1, AR, CD36, EEF1A2, MAPK10, SNCG, TAGLN, NGFR, and NTRK3 were associated with overall survival (Figure S3A–I). In a separate analysis, HPA‐based immunohistochemical data showed higher protein expression of ADAMTSL1, EEF1A2, SNCG, and TAGLN in GC tissues than in normal gastric tissues (Figure S3J). Considering model inclusion, prognostic relevance, protein‐level evidence, literature support, and experimental feasibility, EEF1A2 and TAGLN were selected for functional validation. After siRNA‐mediated knockdown of EEF1A2 or TAGLN in HGC‐27 cells, Western blotting and qRT‐PCR confirmed reduced protein and mRNA expression levels (Figure 5A,B). Transwell assays showed that silencing either gene significantly decreased HGC‐27 cell migration and invasion (Figure 5C,D). These findings suggest that EEF1A2 and TAGLN may contribute to GC cell migration and invasion.

**FIGURE 5:**
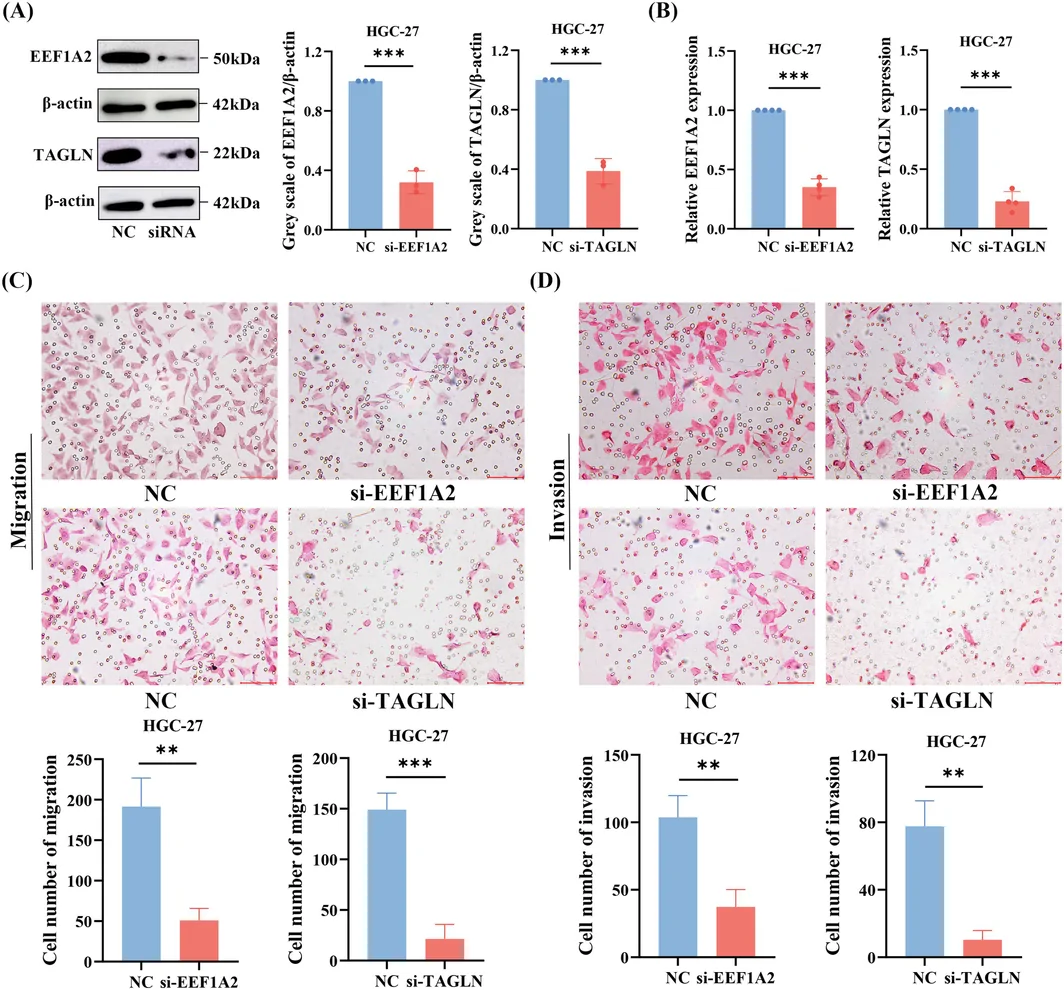
Effects of EEF1A2 and TAGLN knockdown on GC cell migration and invasion. (A) Western blotting and grayscale analysis of EEF1A2 and TAGLN expression and (B) qRT‐PCR analysis of EEF1A2 and TAGLN expression after siRNA‐mediated knockdown in HGC‐27 cells. (C, D) Representative images and quantification of Transwell migration and invasion assays after EEF1A2 or TAGLN knockdown, respectively. Data are presented as mean ± standard deviation; error bars indicate standard deviation, and each dot represents one replicate. Student's *t*‐test was used. **p < 0.01 and ***p < 0.001.

## 4 Discussion

GC remains a leading cause of cancer‐related mortality worldwide (Joshi and Badgwell 2021). Because early symptoms are often nonspecific, many patients are diagnosed at advanced stages and have poor outcomes. Despite advances in chemotherapy, radiotherapy, targeted therapy, and immunotherapy, recurrence and metastasis remain major obstacles (Sundar et al. 2025). Therefore, identifying prognostic biomarkers and molecular features associated with GC progression remains clinically important. In this study, we developed and externally validated an ARG‐based risk model with moderate prognostic discrimination. The model was associated with overall survival, immune infiltration, stromal features, somatic mutational landscapes, and single‐cell‐derived stromal remodeling features. Functional experiments further suggested that EEF1A2 and TAGLN may contribute to GC cell migration and invasion.

Anoikis maintains tissue homeostasis by eliminating detached cells and preventing aberrant cell survival (Mei et al. 2024). Cancer cells may acquire anoikis resistance through pathways such as AMPK and Hippo signaling, allowing survival during circulation and colonization at distant sites (Jin et al. 2018; Shi et al. 2024; Simpson et al. 2008). Previous studies have linked anoikis‐related molecules, including SERPINE1 and glucose‐regulated protein 94, to metastatic potential and TME interactions (Wang et al. 2025; Shi et al. 2024). Consistent with these findings, our results suggest that ARG‐related transcriptional programs are associated with GC prognosis and may be linked to stromal remodeling and EndMT‐associated cellular states. These observations provide a potential context for understanding the relationship between anoikis‐related molecular features and TME remodeling in GC.

Among the core ARGs, EEF1A2 and TAGLN showed higher protein expression in GC tissues and were associated with unfavorable survival outcomes. Knockdown of either gene reduced HGC‐27 cell migration and invasion, supporting their potential involvement in GC progression. Previous studies have linked EEF1A2 and TAGLN to tumor progression and metastasis in multiple malignancies, including GC, through processes related to protein synthesis, cytoskeletal remodeling, and cell motility (Li et al. 2023; Patel et al. 2024; Qiu et al. 2016, 2025). However, these assays evaluated migration and invasion rather than anoikis resistance or EndMT itself. Thus, these findings support the pro‐migratory and pro‐invasive roles of EEF1A2 and TAGLN but do not establish their direct involvement in anoikis resistance or EndMT.

scRNA‐seq analysis revealed cellular heterogeneity of ARG‐related transcriptional programs in GC, with core ARGs showing cell type‐specific expression in endothelial cells and fibroblasts. inferCNV analysis suggested increased chromosome‐scale transcriptional heterogeneity in these stromal populations, whereas pseudotime analysis indicated potential transcriptional‐state relationships between endothelial cells and fibroblast subpopulations. Together with the positive correlation between ARG score and EndMT score, these findings suggest that ARG‐related transcriptional programs may be associated with EndMT‐like stromal features rather than definitive EndMT events. Several ARGs, including CD36, SFRP1, and CRYAB, showed dynamic expression patterns along the inferred stromal‐state trajectory, consistent with previous studies linking these genes to GC progression, stromal remodeling, and metastatic potential (Jacome‐Sosa et al. 2021; Aoki et al. 2023; Jiang et al. 2023; Winkler et al. 2024; Xia et al. 2024). In addition, the positive correlation between the ARG‐based risk score and CD86 expression may reflect increased activity of antigen‐presenting cells, macrophages, or DCs, highlighting the complexity of immune‐stromal interactions in the GC microenvironment.

Several limitations should be acknowledged. The initial ARG collection was based on a broad GeneCards retrieval strategy, and potential noise from the initial gene set cannot be fully excluded despite downstream differential expression, survival, and machine‐learning‐based feature selection analyses. The prognostic performance and clinical applicability of the ARG‐based risk model require further validation in larger, independent, multicenter cohorts. In addition, the functional experiments were performed in a single GC cell line, and dedicated anoikis resistance assays were not conducted. Therefore, the functional findings should be interpreted as preliminary evidence related to GC cell migration and invasion rather than direct evidence of anoikis resistance. Similarly, inferCNV and pseudotime analyses were used to evaluate chromosome‐scale transcriptional heterogeneity and potential transcriptional‐state relationships, not true genomic copy number alterations or definitive EndMT events. These findings should be interpreted as auxiliary transcriptomic evidence.

## 5 Conclusion

In conclusion, this study developed an externally validated ARG‐based risk model associated with prognosis, TME features, and somatic mutational landscapes in GC. Single‐cell and in vitro analyses further suggested that ARG‐related transcriptional programs may be linked to EndMT‐associated stromal remodeling and GC cell migration and invasion. These findings support the clinical and biological relevance of ARG‐related molecular features in GC.

## Author Contributions

**Zhijun Fu:** conceptualization, methodology, formal analysis, data curation, investigation, writing – original draft. **Lin Sun:** data curation, formal analysis, validation, writing – review and editing. **Xing Wang:** investigation, resources, validation, visualization. **Biao Cheng:** writing – review and editing, data curation. **Ying Huang:** data curation, writing – review and editing. **Yuzhu Tang:** data curation, writing – review and editing. **Zhihua Jin:** conceptualization, supervision, resources, project administration, writing – review and editing.

## Funding

The authors have nothing to report.

## Conflicts of Interest

The authors declare no conflicts of interest.

## Supporting information


**Figure S1:** Quality assessment, doublet identification, cell‐cycle analysis, and principal component analysis of scRNA‐seq data.
**Figure S2:** Doublet distribution and pseudotime expression analysis in scRNA‐seq data.
**Figure S3:** Survival analysis and protein expression validation of core ARGs.
**Table S1:** Complete list of 913 anoikis‐related genes (ARGs) retrieved from the GeneCards database using the keyword “Anoikis‐Related”, including GeneCards identifiers and relevance scores.
**Table S2:** Gene Ontology (GO) enrichment analysis results of the 45 differentially expressed ARGs identified in the TCGA‐STAD cohort.
**Table S3:** Kyoto Encyclopedia of Genes and Genomes (KEGG) enrichment analysis results of the identified differentially expressed ARGs.
**Table S4:** C‐index values of 101 machine‐learning algorithm combinations evaluated in the TCGA‐STAD and GSE84437 cohorts.
**Table S5:** Univariate and multivariate Cox proportional hazards regression analyses of clinicopathological variables and ARG‐based risk score in the TCGA‐STAD cohort.

## Data Availability

The datasets analyzed in this study are publicly available. Transcriptomic and clinical data for The Cancer Genome Atlas Stomach Adenocarcinoma (TCGA‐STAD) cohort were obtained from the UCSC Xena platform. The GSE84437 and GSE162115 datasets were downloaded from the Gene Expression Omnibus (GEO) database. Anoikis‐related genes were retrieved from the GeneCards database. Immunohistochemical data were obtained from the Human Protein Atlas (HPA). Whole‐exome sequencing (WES)‐derived somatic mutation data for TCGA‐STAD were obtained from cBioPortal. Additional data supporting the findings of this study are available from the corresponding author upon reasonable request.
