# Supplementary material for: An Anoikis‐Related Gene Signature Associated With Stromal Remodeling and Clinical Outcome in Gastric Cancer
Source: Chem Biol Drug Des. 2026 Sep 30;108(4):e70411. doi: 10.1111/cbdd.70411 (PMC13627849; doi:10.1111/cbdd.70411)
Supplement: Supplementary file 1 [file CBDD-108-e70411-s001.zip › ARG_GC_Supplementary_Materials_R2/ARG_GC_Supplementary_Figures_R2/ARG_GC_Supplementary Figure Legends.docx]

**Supplementary Figure 1. Quality assessment, doublet identification, cell-cycle analysis, and principal component analysis of scRNA-seq data.**

(A) Distribution of nFeature_RNA and nCount_RNA across different samples.

(B) Identification of predicted doublets and singlets using the DoubletFinder algorithm and t-SNE visualization.

(C) Violin plots of nCount_RNA and nFeature_RNA in predicted doublets and singlets.

(D) Heatmaps of representative genes associated with the first four principal components (PC1–PC4).

(E) Cell-cycle scoring analysis based on G1-, S-, and G2/M phase-related genes and t-SNE visualization of cell-cycle states.

**Supplementary Figure 2. Doublet distribution and pseudotime expression analysis in scRNA-seq data.**

(A) t-SNE visualization of predicted singlets and doublets identified during single-cell quality control.

(B) Expression of CD36, CRYAB, and SFRP1 along the pseudotime trajectory.

**Supplementary Figure 3. Survival analysis and protein expression validation of core ARGs.**

(A–I) Kaplan–Meier survival curves of nine core ARGs in GC based on the TCGA-STAD cohort.

(J) Immunohistochemical staining images of ADAMTSL1, EEF1A2, SNCG, and TAGLN in normal gastric tissues and GC tissues obtained from the HPA.
