# Supplementary material for: An Anoikis‐Related Gene Signature Associated With Stromal Remodeling and Clinical Outcome in Gastric Cancer
Source: Chem Biol Drug Des. 2026 Sep 30;108(4):e70411. doi: 10.1111/cbdd.70411 (PMC13627849; doi:10.1111/cbdd.70411)
Supplement: Supplementary file 1 [file CBDD-108-e70411-s001.zip › ARG_GC_Supplementary_Materials_R2/ARG_GC_Supplementary_Tables_R2/ARG_GC_Supplementary_Table_Legends.docx]

**Supplementary Table Legends**

**Table S1.** Complete list of 913 anoikis-related genes (ARGs) retrieved from the GeneCards database using the keyword “Anoikis-Related”, including GeneCards identifiers and relevance scores.

**Table S2.** Gene Ontology (GO) enrichment analysis results of the 45 differentially expressed ARGs identified in the TCGA-STAD cohort.

**Table S3.** Kyoto Encyclopedia of Genes and Genomes (KEGG) enrichment analysis results of the identified differentially expressed ARGs.

**Table S4.** C-index values of 101 machine-learning algorithm combinations evaluated in the TCGA-STAD and GSE84437 cohorts.

**Table S5.** Univariate and multivariate Cox proportional hazards regression analyses of clinicopathological variables and ARG-based risk score in the TCGA-STAD cohort.
